# The Six-Transmembrane Epithelial Antigen of the Prostate (STEAP) 3 Regulates the Myogenic Differentiation of Yunan Black Pig Muscle Satellite Cells (MuSCs) In Vitro via Iron Homeostasis and the PI3K/AKT Pathway

**DOI:** 10.3390/cells14090656

**Published:** 2025-04-29

**Authors:** Wei Zhang, Minying Zhang, Jiaqing Zhang, Sujuan Chen, Keke Zhang, Xuejing Xie, Chaofan Guo, Jiyuan Shen, Xiaojian Zhang, Huarun Sun, Liya Guo, Yuliang Wen, Lei Wang, Jianhe Hu

**Affiliations:** 1 Henan Institute of Science and Technology, College of Animal Science and Veterinary Medicine, Xinxiang 453003, China; yuntaishan2003@aliyun.com (W.Z.); 15903847294@163.com (M.Z.); a2142523295@163.com (K.Z.); 15136753082@163.com (X.X.); 18839119906@163.com (C.G.); shenjy@hist.edu.cn (J.S.); zxj9109@126.com (X.Z.); sunhuarun@hist.edu.cn (H.S.); nzzc2008@126.com (L.G.); yuliang_wen@163.com (Y.W.); 2 Institute of Animal Husbandry, Henan Academy of Agricultural Sciences, Zhengzhou 450002, China; zhangjq@hnagri.org.cn; 3 School of Life Sciences and Technology, Xinxiang Medical University, Xinxiang 453003, China; chensujuan101@163.com

**Keywords:** STEAP3, MuSCs myogenic differentiation, iron homeostasis, PI3K/AKT pathway

## Abstract

The myogenic differentiation of muscle satellite cells (MuSCs) is an important biological process that plays a key role in the regeneration and repair of skeletal muscles. However, the mechanisms regulating myoblast myogenesis require further investigation. In this study, we found that STEAP3 is involved in myogenic differentiation based on the Yunan black pig MuSCs model in vitro using cell transfection and other methods. Furthermore, the expression of myogenic differentiation marker genes *MyoG* and *MyoD* and the number of myotubes formed by the differentiation of cells from the si-STEAP3 treated group were significantly decreased but increased in the STEAP3 overexpression group compared to that in the control group. STEAP3 played a role in iron ion metabolism, affecting myogenic differentiation via the uptake of iron ions and enhancing IRP-IRE homeostasis. STEAP3 also activated the PI3K/AKT pathway, thus promoting myoblast differentiation of Yunan black pig MuSCs. The results of this study showed that STEAP3 overexpression increased intracellular iron ion content and activated the homeostatic IRP-IRE system to regulate intracellular iron ion metabolism.

## 1. Introduction

As primary body tissues, skeletal muscles play key roles in locomotion, postural maintenance, respiration, and thermogenesis in humans. They are also involved in the slaughter performance of animal breeds, which heavily depend on the regeneration and repair of skeletal muscle [1,2,3]. The key step in this complex process is the differentiation of myoblasts into post-mitotic myocytes and proliferative myoblasts, followed by fusion with pre-existing myotubes to form repaired muscle fibers [4,5]. MyoD expression can induce skeletal muscle differentiation as a marker in myoblasts derived from mesodermal derivation and in some cell types derived from the two other germ layers [6,7]. Myogenic differentiation is regulated by a series of cellular signaling pathways, including those involving protein kinases—which are initiated by promyogenic stimuli—and the reversible phosphorylation process that executes the signals. Furthermore, the PI3K/AKT pathway appears to act as an effector of different factors that can generate myogenic differentiation under stage-specific conditions [8,9]. For instance, glucagon-like peptide-2 (GLP-2) significantly reverses the decline in muscle weight, relative grip strength, diameter, and cross-sectional area of muscle fibers in aging mice by activating the IGF-1/PI3k/Akt/FoxO3a phosphorylation pathway [10]. In addition, 4-octyl itaconate (OI) inhibits muscle differentiation by affecting MyoD-regulated activity through the inhibition of PI3K/AKT [11]. Although the PI3K/Akt pathway promoted myogenic differentiation is well documented, many of its exact mechanisms remain to be identified [12].

The STEAP family, including STEAP1, STEAP2, STEAP3, and STEAP4, belongs to the transmembrane protein family. It shares structural features containing six transmembrane structural domains, all of which act as metal reductases in vivo [13,14,15,16,17,18,19]. The STEAP family has ion channel functions, can exert metal oxidoreductase activity in vivo, and is widely involved in regulating cell proliferation, apoptosis, migration, invasion, and other cellular processes. Despite their similar structures, different STEAPs have specific expression patterns and are expressed differently in individual tissues under different conditions [20,21,22]. STEAP3 is a 488-amino-acid (54.6 kDa) protein and is involved in various biological processes, including ferroptosis, apoptosis, proliferation, and inflammation [23,24,25]. In particular, STEAP3 consists of a hexameric transmembrane structure in the C-terminal domain and an oxidoreductase structure in the N-terminal domain. This is important in regulating iron homeostasis by reducing trivalent iron to divalent iron in vivo [13]. Studies on STEAP3 have focused on its roles in cancer and cellular immunity. For instance, STEAP3 is overexpressed in the nucleus of hepatocellular carcinoma cells [26] and regulates ferroptosis through the p53/SLC7A11 pathway during ovarian cancer progression [27]. STEAP3 is also a potential candidate biomarker for several cancers and a potential target for new immunotherapeutic strategies for disease attenuation or treatment [18]. However, STEAP3, an iron reductase enzyme, is an essential component of the transferrin–transferrin receptor cycle and intracellular iron homeostasis and colocalizes with transferrin, transferrin receptor, and DMT1 during erythroid–transferrin endosome-mediated iron uptake. In addition, STEAP3 was expressed in hematopoietic tissues and supports important physiologic functions related to iron metabolism, especially in erythroid precursors [25,28,29]. Interestingly, STEAP3 has also been detected in skeletal muscles [28,30]. However, there is a lack of information on the expression pattern, physiological functions, and unique mechanism of STEAP3 in skeletal muscle development and the regulation of myoblast myogenesis, which requires further study.

Iron is an essential micronutrient that is vital for both life and health. It is an important part of the synthesis of hemoglobin, myoglobin, and various enzymes involved in oxygen transport, DNA synthesis, ATP synthesis, and other life activities [31,32]. Iron, belonging to the transition metal elements, has unique electrochemical properties. It can readily accept and donate electrons and switch between the Fe (II) and Fe (III) valence states in intracellular biological processes, including ATP generation, DNA synthesis and repair, and oxygen transport [33,34,35]. However, the balanced metabolism of iron is essential to various biological processes. It has been demonstrated that too low or too high levels of intracellular iron can reduce the normal functions of cells, even inducing ferroptotic cell death [36,37]. The maintenance of the homeostasis of intracellular iron metabolism mainly involves regulating iron uptake, homeostasis, and exocytosis to balance the needs of the organism. Mammalian cells absorb iron ions mainly through transferrin binding and endocytosis into the cell via the binding of TfRl to the cell membrane [38,39]. Cellular iron homeostatic metabolism is mainly regulated by iron regulatory proteins (IRPs), which are categorized into IRP1 and IRP2, and play a role in RNA binding to regulate the uptake and metabolism of intracellular iron ions when there is an imbalance in intracellular iron metabolism [34,35,40]. STEAP3 reduces Fe^3+^ entering the cell to Fe^2+^ for various physiological activities of the cell, and the excess iron ions are stored in ferritin during this process [39,41]. Recent studies have shown that the process of myogenic differentiation of myoblasts also requires iron ions. Researchers created an iron-deficient environment during the culture of the mouse myoblast cell line C2C12 and found that myoblasts have decreased proliferation and differentiation ability when they are iron deficient [42,43]. Furthermore, as the first new national breed of livestock in Henan Province, the Yunan black pig, a crossbreed between the Huainan Pig—an ancient pig breed in northern China with lower growth speed—and the duroc pig as a male parent, is famous for its good meat quality and fast growth. After breeding for many years, the Yunan black pig showed notable slaughter weight and slaughter performance compared to that of Huainan pigs [44,45]. Consumers prefer it because of its unique organoleptic characteristics, influenced by lipids and volatiles [46,47]. Therefore, Yunan black pigs were used as a suitable model to study myoblast myogenic differentiation to improve growth speed. Further studies on the mechanism of action of STEAP3 and iron in skeletal myoblast myogenesis must be conducted based on the Yunan black pig model.

In this study, to make sure of the exact role and mechanism of STEAP3 during myogenic differentiation of Yunan black pig MuSCs, we firstly obtained it in vitro. We found that the high expression of STEAP3, the increase of Fe^2+^ during cell differentiation, intracellular iron overload, and iron depletion affect myoblast differentiation. Therefore, we speculated that STEAP3 regulates myoblast differentiation mainly through regulating intracellular iron ions via the PI3K/AKT pathway.

## 2. Materials and Methods

### 2.1. The Skeletal MuSCs Isolation, Cell Culture, and Muscle Sample Collection

The MuSCs used in this experiment were isolated from the thigh skeletal muscle tissues of 3-day-old Yunan black pigs according to the methods described in a previous study [48]. Briefly, 3-day-old piglets (*n* = 3) were euthanized via intravenous injection of 20% sodium pentobarbital (EUTHASOL, 500 mg/mL) [49,50]. Then, we collected the skeletal muscle tissues from the thighs, preserved them in phosphate-buffered saline (PBS) containing a double-antibiotic solution, and dissected the tissues. The adult pigs of 6 m (*n* = 3) were euthanized under general anesthesia following the usual protocol (midazolam 15 mg, chlorpromazine 25 mg, KCL 15% (*w*/*v*) 20 mL) [51,52]. Then, 1 g of muscle close to the thigh skeletal muscle was removed and cleaned in sterile PBS (Solarbio, Beijing, China, P1020). It was cut into pieces, and 3–4 times the volume of tissue was added to 0.2% collagenase II (Solarbio, Beijing, China, C8150), digested at 37 °C for 30 min, and centrifuged at 1500 rpm for 10 min. Subsequently, 5 mL of 0.25% pancreatic enzyme (Solarbio, Beijing, China, T1320) was added and incubated for 20 min to digest. An equal amount of DMEM/F12 (Solarbio, Beijing, China, D6501) was added to terminate the digestion, and the tissue solution was sequentially passed through 200 mesh and 400 mesh cell sieves (Solarbio, Beijing, China, YA0949, YA0951). The supernatant was discarded. The tissue solution was sequentially passed through 200 mesh and 400 mesh cell sieve, followed by centrifugation at 1500 rpm for 10 min. The supernatant was discarded and cultured in DMEM/F12 medium supplemented with 10% fetal bovine serum (FBS, Yeasen, Shanghai, China, 40130ES76) and 1% Penicillin-streptomycin (Yeasen, Shanghai, China, 60162ES76). When the confluence reached about 80–90%, the growth medium was replaced with differentiation medium containing DMEM, 2% horse serum (Thermo Fisher, Beijing, China, 26050070), and 2% penicillin/streptomycin in order to induce MuSC differentiation [53]. All cells were cultured in a 37 °C incubator at 5% CO_2_. Primary myoblast cells were treated with siRNA or overexpression plasmids and cultured in a growth medium. To inhibit PI3K, 20 μM LY294002 (Beyotime, Shanghai, China, S1737) was added to the interfering or overexpressed cells 24 h later. After cells reached 100% confluence, the cells were switched to differentiation medium to induce differentiation for 1–5 days. Sample isolation was performed according to the Animal Care and Use Statute of China and was approved by the Animal Care and Use Committee of the Henan Institute of Science and Technology (No. 2024667).

### 2.2. Identification of Yunan Black Pig MuSCs

Isolated cells were inoculated into six-well plates, and cell identification was performed when the cells grew to approximately 85%. The cultured cells were fixed with paraformaldehyde (Biosharp, Hefei, China, BL539A) for 15 min, permeabilized with 0.5% Triton-100 (Beyotime, Shanghai, China, P0096) for 15 min, closed with 1% BSA (Solarbio, Beijing, China, SW3015) sealing solution for 1 h, and then washed twice with PBS. Thereafter, it was incubated overnight with desmin (Proteintech, Wuhan, China, 16520-1-AP, 1:300) primary antibody. The primary antibody was recovered, and the wells were washed three times with PBST (Solarbio, Beijing, China, P1033) and incubated with the secondary antibody (Proteintech, Wuhan, China, B900210, 1:1000) for 1 h in the dark. The nuclei of the cells were stained with DAPI (Elabscience, Wuhan, China, E-CK-A163) for 5 min, and finally, a drop of anti-fluorescence quencher (Solarbio, Beijing, China, S2110) was added. Sealed slices were observed under a fluorescence microscope.

### 2.3. Immunohistochemistry

According to our previous studies, porcine skeletal muscle tissue sections were deparaffinized by xylene and gradient alcohol, and then antigenically repaired (ZSGB-Bio, Beijing, China, ZLI-9067). Endogenous peroxidase was blocked using 3% hydrogen peroxide (ZSGB-Bio, Beijing, China, 7722-84-1), after which 3% BSA (Solarbio, Beijing, China, SW3015) was used to seal the sections for 30 min at room temperature. The sealing solution was removed and the primary antibody STEAP3 (NOVUS, Shanghai, China, NBP1-76824, 1:200) was added and incubated overnight in a wet box at 4 °C. The primary antibody was washed off and the secondary antibody was added and incubated at room temperature for 1 h. DAB color development solution (ZSGB-Bio, Beijing, China, ZLI-9017) was added, and finally, hematoxylin (Solarbio, Beijing, China, G1080) was used for re-staining. The film was sealed after gradient alcohol dehydration and observed under the microscope [54,55].

### 2.4. RNA Isolation and qRT-PCR

Total RNA from the samples was extracted using TRIzol reagent (TAKARA, Beijing, China, 9180). cDNAs was synthesized using HiScript III RTSuperMix (TAKARA, Beijing, China, RR037A) according to the supplier’s protocol. The gene expression was detected using primer sequences (Table 1) and MonAmp SYBR Green qPCR Mix (TAKARA, Beijing, China, RR420A) on a QuantStudio 5 Flex Real-Time PCR System (Thermo Fisher, Beijing, China).

### 2.5. Cell Transfection

Three siRNAs targeting STEAP3 were purchased from Sangon Biotech, Shanghai, China. The best siRNA sequences were as follows. The sense strand sequences: 5′-UAGCUUGUAGAACUUGUUCTT-3′. The antisense strand sequences: 5′-GAACAAGUUCUACAAGCUATT-3′. For the STEAP3 overexpression vector, the coding sequences (CDSs) of the porcine *STEAP3* gene were inserted into the pEGFP-N1 vector (Sangon Biotech, Shanghai, China). Primary myoblast cells were seeded into six-well plates until those cell confluence reached 70–80% when they were transfected with siRNAs using Lipofectamine 3000 (Thermo Fisher, Beijing, China, L3000015). After 6 h of transfection, the cells were washed once with PBS and incubated with DMEM containing 10% FBS or differentiation medium to maintain cell proliferation and induce the formation of myotubes, respectively.

### 2.6. Immunofluorescence Staining

Primary myoblast cells and the cells from the 3rd and 5th days of myogenic differentiation were fixed with 4% paraformaldehyde (Biosharp, Hefei, China, BL539A) for 15 min, permeabilized in 0.5% Triton X-100 (Beyotime, Shanghai, China, P0096) for 15 min, and blocked with 1% BSA (Solarbio, Beijing, China, SW3015) for 1 h. The primary antibody MyHC (Priteintech, Wuhan, China, 22281-1-AP, 1:500) was added and incubated overnight, followed by the addition of the corresponding secondary antibody, and incubated for 2 h. Subsequently, DAPI (Elabscience, Wuhan, China, E-CK-A163) was added to stain the nuclei for 5 min. Images were captured using a fluorescence microscope (Nikon, Tokyo, Japan). The differentiation rate was calculated as the percentage of nuclei in MyHC-positive cells. Nine immunofluorescence images from three replicates (three from each replicate) were randomly selected for analysis in each group.

### 2.7. Protein Isolation and Western Blot Analyses

Total proteins were extracted from whole-cell lysates or skeletal muscles using RIPA Lysis Buffer (Beyotime, Shanghai, China, P0013B). After centrifugation at 12,000× *g* at 4 °C for 10 min, the supernatant was obtained and the protein concentration was determined using a BCA assay kit (Beyotime, Shanghai, China, P0011). The protein samples (equal amounts) were added to the loading buffer and incubated at 100 °C for 10 min. Then, 20 μL protein samples were separated using 8–15% SDS–PAGE at 80 V and transferred to polyvinylidene fluoride membranes (PVDF, Immobilon, Shanghai, China, IPVH00010). Then, membranes were blocked with 5% milk (Solarbio, Beijing, China, D8340) in PBST for 1 h at room temperature and incubated overnight. The next day, the membranes were washed three times with PBST and incubated with secondary antibodies for 1 h. Finally, the membranes were exposed to ECL luminescent solution (Yeasen, Shanghai, China, 36208ES60), and protein grayscale analysis was performed using the Image J software (v1.54d). The primary and secondary antibodies and their dilution ratios used were as follows: STEAP3 (Proteintech, Wuhan, China, 28478-1-AP, 1:2000), PI3K (Proteintech, Wuhan, China, 60225-1-Ig, 1:5000), P-PI3K (ABMART, Shanghai, China, T40064, 1:3000), AKT (Proteintech, Wuhan, China, 10176-2-AP, 1:5000), P-AKT (Proteintech, Wuhan, China, 66444-1-Ig, 1:3000), β-actin (ABMART, Shanghai, China, P30002, 1:5000), and β-actin (Proteintech, 66009-1-Ig, 1:10,000).

### 2.8. Cell Iron Pool Detection

Calcein-AM (Yeasen, Shanghai, China, 40719ES60) combined with iron pool quenching fluorescence was used to detect the cell iron pool content. The treated myoblasts were collected and washed twice with flow cytometry loading buffer (Proteintech, Wuhan, China, PF00018), then incubated at 37 °C with complete medium containing 0.1 µM Calcein-AM for 30 min. The cell pellet was collected and washed twice. The fluorescence intensity was determined using a FACSCalibur flow cytometer (BD Bioscience, Franklin Lakes, NJ, USA) and analyzed using FlowJo 10.8.1.

### 2.9. Cell Ferrous Iron Colorimetric Assay

A Cell Ferrous Iron Colorimetric Assay Kit (Elabscience, Wuhan, China, E-BC-K881-M) was used to determine intracellular ferrous iron levels. Approximately 1 × 10^6^ cells were extracted and homogenized on ice for 10 min with 200 μL of lysis buffer before being centrifuged at 15,000× *g* for 10 min to collect the supernatant. Then, 80 μL of supernatant was treated with the iron probe or the control reagent for 10 min at 37 °C. The intracellular ferrous ion content was obtained using the absorbance value at 593 nm of the enzyme marker and substituting the measured standard curve.

### 2.10. Statistical Analysis

Continuous variables were expressed as the mean ± standard error of the mean (SEM). Statistical analysis was performed using a commercially available program, Statistical Package for the Social Sciences (SPSS Version 18.0; Chicago, IL, USA). Independent sample *t*-test is used for the comparison between two groups, and one-way analysis of variance (One-way ANOVA) is used for the statistical analysis of data among multiple groups. * *p* < 0.05, ** *p* < 0.01, *** *p* < 0.001.

## 3. Results

### 3.1. Isolation, Myogenic Differentiation, and Identification of Yunan Black Pig MuSCs

**Figure 1:**
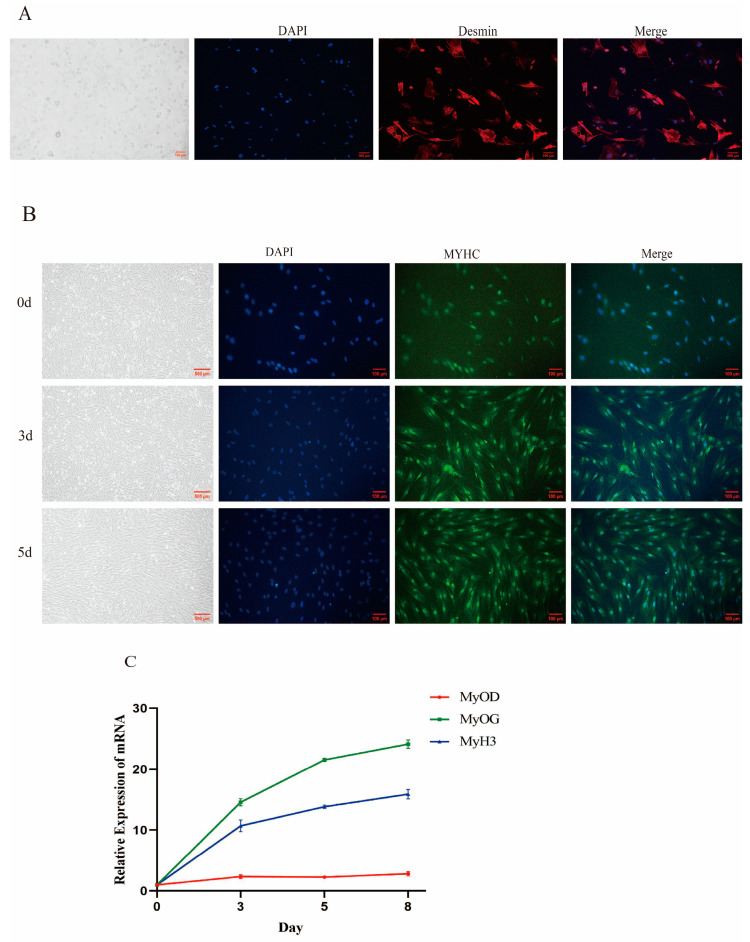
Isolation, myogenic differentiation and identification of YuNan black pig MSCs. (**A**) Identification of porcine primary skeletal muscle satellite cells (Bar: 100 μm). (**B**) Induced differentiation of porcine primary skeletal muscle satellite cells (Bar: 500 μm, 100 μm). (**C**) Detection of marker genes for myogenesis. *n* = 3.

We used enzymatic digestion and differential culture to isolate pig MuSCs. The MuSCs observed under a light microscope were single, free, round, and transparent. After cell immunofluorescence, the isolated cells were positively reacted with primary anti-myosin (desmin) after fixation, and the purity of cells was >95%. After being induced for 5 days, immunofluorescence staining of the myotube differentiation marker gene *MyHC* showed that the cells were long, shuttle-shaped, and arranged in parallel (Figure 1A). The qPCR results showed that the expression of *MyoD*, *MyoG*, and *MyH3* increased significantly with an increase in differentiation days (*p* < 0.01) (Figure 1B), which proved that the cells were well differentiated. In summary, MuSCs were successfully isolated from Yunan black pigs and an in vitro differentiation model was established.

### 3.2. Expression, Distribution, and Role of STEAP3 in MuSCs and Muscle Tissue

**Figure 2:**
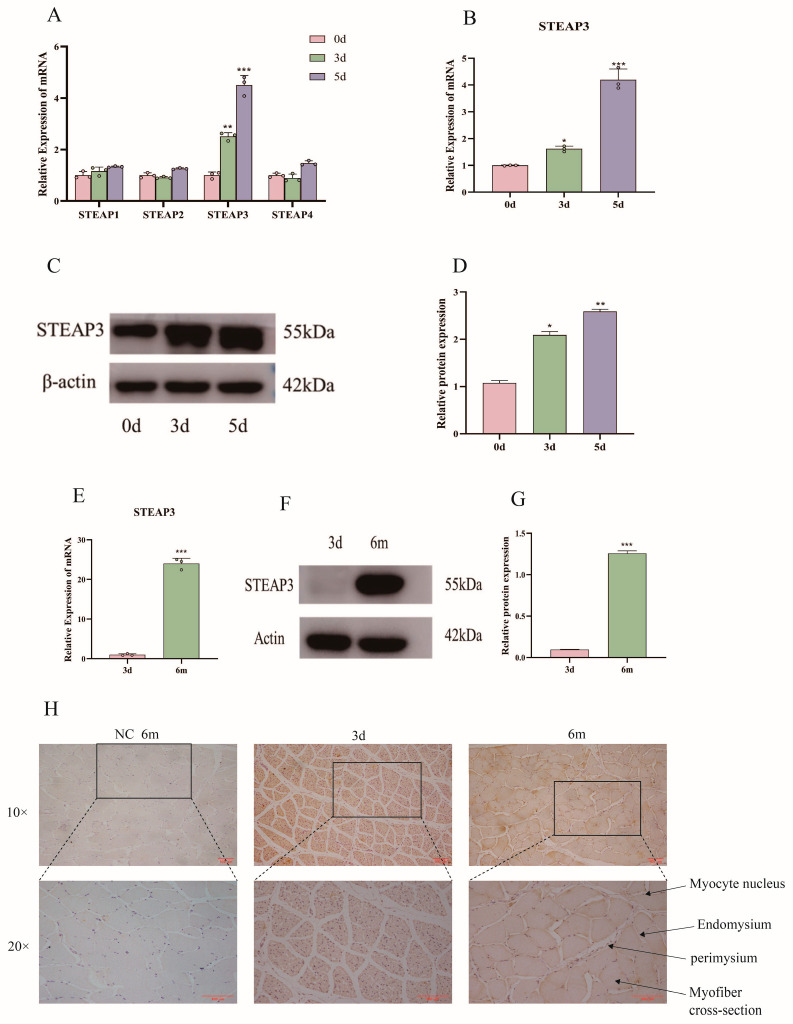
Expression, distribution, and role of STEAP3 in primary muscle satellite cells and muscle tissue. (**A**) mRNA expression of STEAP family during differentiation. (**B**) mRNA expression of *STEAP3* during differentiation. (**C**,**D**) Protein expression of STEAP3 during differentiation. (**E**) mRNA expression of *STEAP3* in skeletal muscle of pigs of different ages. (**F**,**G**) Protein expression of STEAP3 in skeletal muscle of pigs of different ages. (**H**) Distribution of STEAP3 in skeletal muscle of pigs of different ages (Bar: 100 μm). *n* = 3, * *p* < 0.05, ** *p* < 0.01, *** *p* < 0.01.

We used qPCR and Western blotting to detect and determine the expression changes in the STEAP family genes. The results showed that STEAP3 expression changed most significantly during differentiation (*p* < 0.001) (Figure 2A). While the mRNA and protein expression of STEAP3 increased significantly with an increase in the number of differentiation days (*p* < 0.001) which indicated that STEAP3 might be involved in regulating myogenesis differentiation of MuSCs (Figure 2B–D). The qPCR (Figure 2E) and Western blotting (Figure 2F,G) results showed that the expression of STEAP3 in skeletal muscle tissues at 6 months was significantly higher than that of the piglets at 3 days (*p* < 0.001). Meanwhile, skeletal muscle IHC showed that the expression of STEAP3 was mainly distributed in the myofibril cell membrane, and the distribution of STEAP3 was significantly higher in the skeletal muscle at 6 months (Figure 2H).

### 3.3. STEAP3 Promotes Yunan Black Pig MuSCs’ Myogenic Differentiation In Vitro

**Figure 3:**
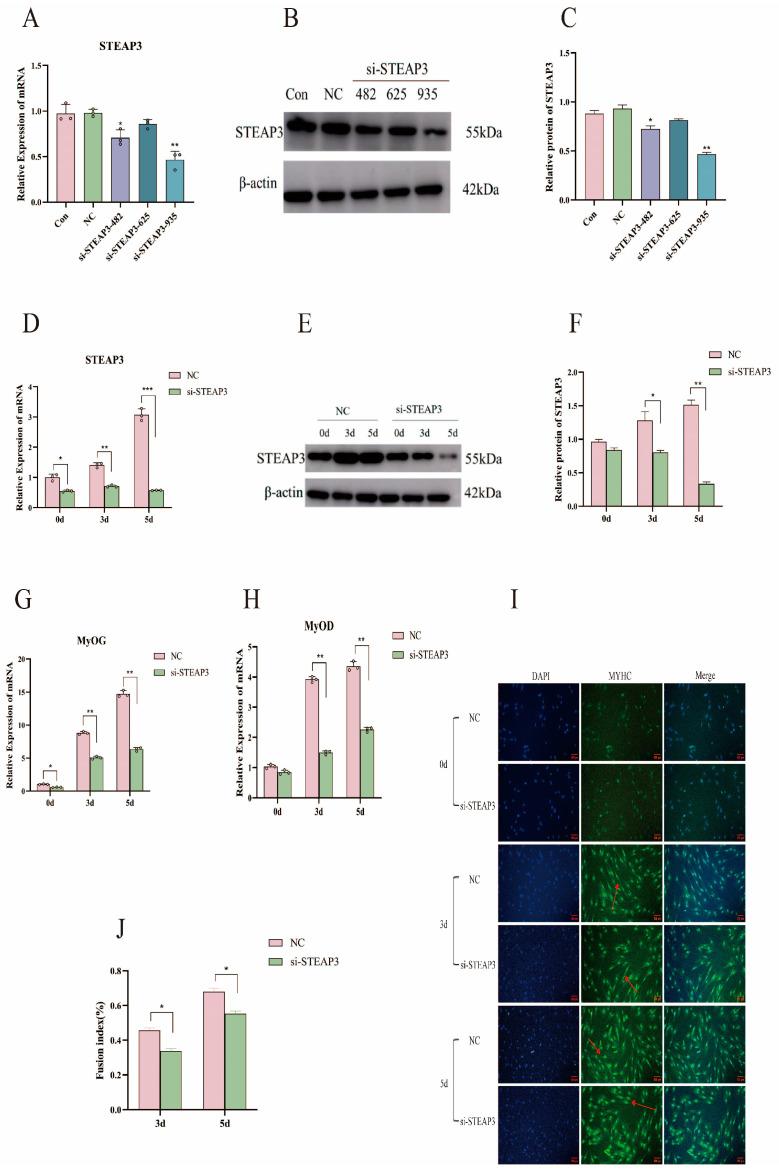
Si-STEAP3 on myogenic differentiation. (**A**) si-STEAP3 sequence screening mRNA detection (1d). (**B**,**C**) si-STEAP3 sequence screening protein detection (1d). (**D**) Interference effect of *STEAP3* during differentiation, mRNA verification. (**E**,**F**) Interference effect of STEAP3 during differentiation, WB verification. (**G**,**H**) Differentiation marker gene detection. (**I**,**J**) Myotube MYHC immunofluorescence detection and confluency. The red arrow points to the myotubes formed after myogenic differentiation (Bar: 100 μm). *n* = 3, * *p* < 0.05, ** *p* < 0.01, *** *p* < 0.01.

**Figure 4:**
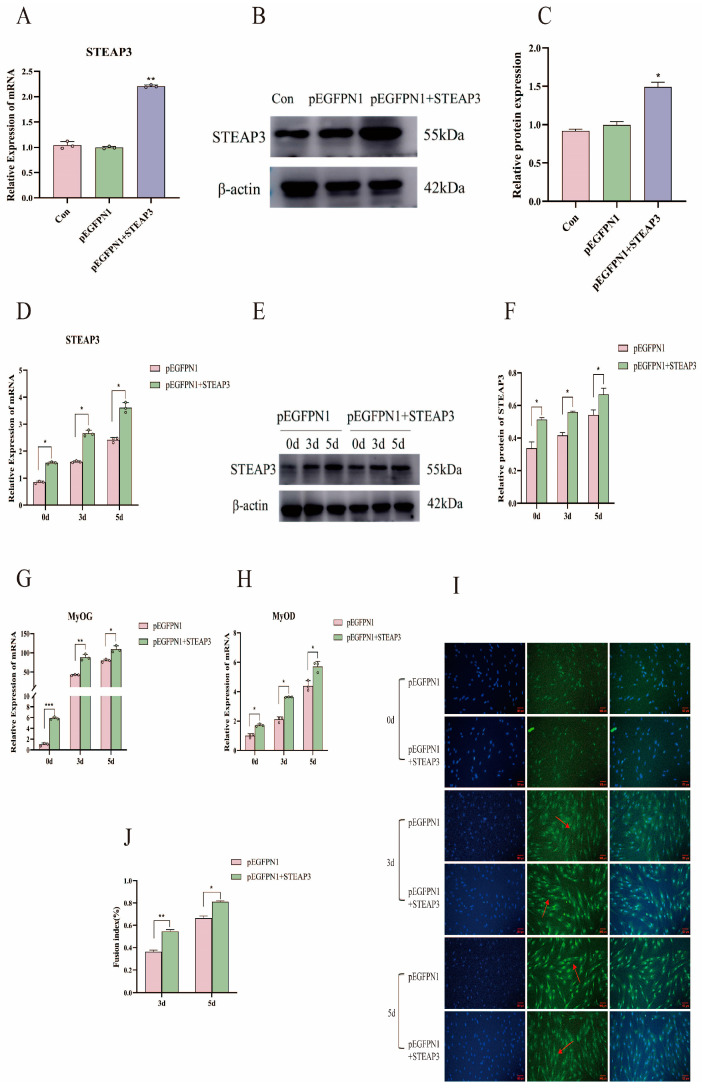
Effect of STEAP3 overexpression on myogenic differentiation. (**A**) plasmid overexpression mRNA detection (1d). (**B**,**C**) Plasmid overexpression protein detection (1d). (**D**) mRNA verification of *STEAP3* overexpression effect during differentiation. (**E**,**F**) Overexpression effect of STEAP3 during differentiation WB verification. (**G**,**H**) Differentiation marker gene detection. (**I**,**J**) Myotube MYHC immunofluorescence detection and confluency. The red arrow points to the myotubes formed after myogenic differentiation (Bar: 100 μm). *n* = 3, * *p* < 0.05, ** *p* < 0.01, *** *p* < 0.01.

Based on the above results, we hypothesized that there is a positive relationship between STEAP3 and MuSC differentiation. We used a cell transfection method to transfect siRNA and STEAP3 overexpression plasmids to test this hypothesis. First, harvesting the cells 1 day after transfection, si-STEAP3-935, with the best interference efficiency, was screened out (*p* < 0.01) (Figure 3A–C) and verified during the differentiation process (*p* < 0.001) (Figure 3D–F). Among them, Con is the complete blank control group, and NC is the siRNA negative control group. Simultaneously, qPCR results showed that the expression of the myogenic differentiation marker genes *MyOG* and *MyOD* was significantly decreased in siRNA-treated MuSCs compared to that in the NC controls (*p* < 0.01) (Figure 3G,H). Immunofluorescence staining of the myotube marker protein MyHC showed a decrease in the number of myotubes formed by the differentiation of cells transfected with si-STEAP3 (*p* < 0.05) (Figure 3I,J). Conversely, when transfected with the STEAP3 overexpression plasmids and the cells also harvested 1 day after transfection (*p* < 0.01) (Figure 4A–C) and validated using qPCR and Western blotting during differentiation, STEAP3 was highly expressed in Yunan black pig MuSCs (*p* < 0.05) (Figure 4D–F), and the expression levels of *MyoG* and *MyoD* increased (*p* < 0.001) (Figure 4G,H). Additionally, high STEAP3 expression led to enhanced differentiation of MuSCs, and immunofluorescence staining for MyHC showed a significant increase in the number of multinucleated myotubes (*p* < 0.05) (Figure 4I,J). Collectively, these results indicated that STEAP3 promotes MuSC differentiation.

### 3.4. STEAP3 Regulated Iron Ions in Regulating Myogenic Differentiation via Homeostatic

**Figure 5:**
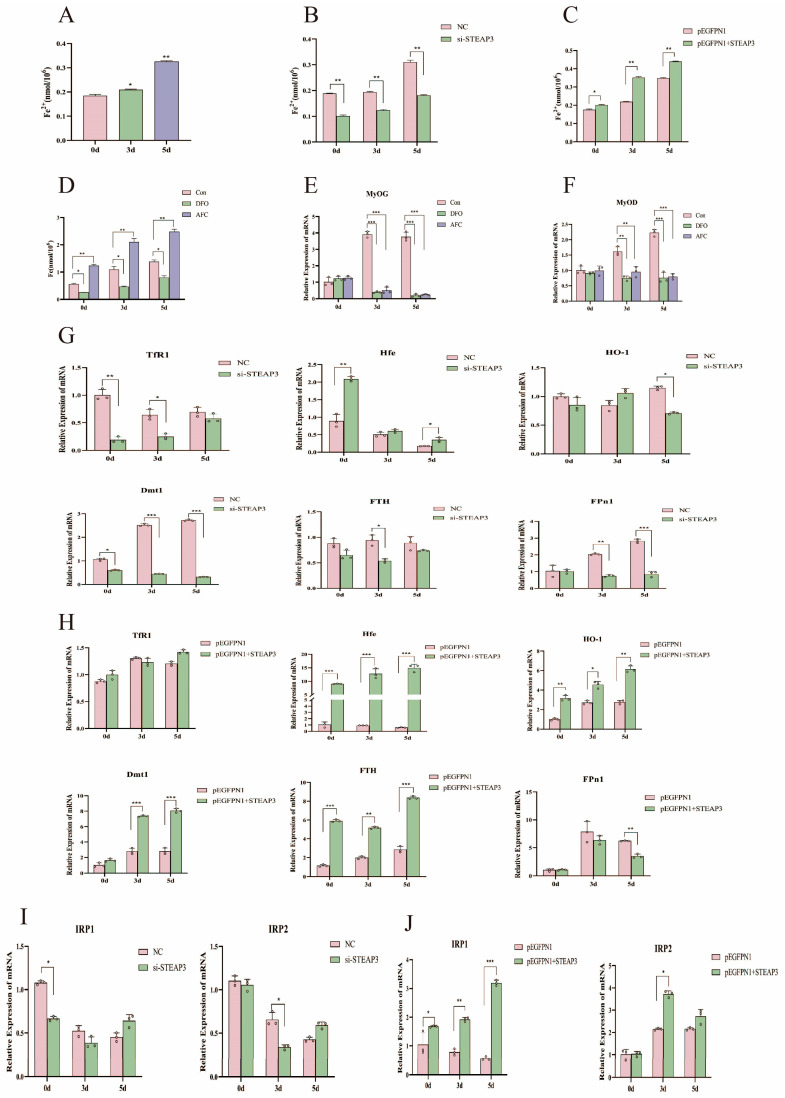
STEAP3 regulated iron ions in the regulation of myogenic differentiation via homeostasis. (**A**) Changes in iron ions during cell differentiation. (**B**) Changes in iron ions when interfering with STEAP3. (**C**) Changes in iron ions on overexpression of STEAP3. (**D**) Changes in iron ions after the addition of DFO and AFC. (**E**,**F**) Genetic testing for markers of myogenesis differentiation. (**G**) Changes in iron metabolism genes when STEAP3 is interfered with. (**H**) Changes in iron metabolism genes in overexpression of STEAP3. (**I**,**J**) Detection of genes of the main pathways of iron metabolism when interfering with and overexpressing STEAP3. *n* = 3, * *p* < 0.05, ** *p* < 0.01, *** *p* < 0.01.

As a metalloreductase, STEAP3 can reduce trivalent iron to divalent iron to be utilized by the organism for growth. We measured the ferrous ion content after different treatments to study the effect of iron ions on myogenic differentiation. The results of this study showed that the intracellular Fe^2+^ content increased significantly with an increase in the number of days of differentiation (*p* < 0.01) (Figure 5A) and intracellular ferrous ions showed the same changes when interfering with or overexpressing STEAP3 (*p* < 0.05) (Figure 5B,C). By exogenously adding AFC (100 µM) and DFO (100 µM) to create an environment of iron overload or iron depletion for the cells (Figure 5D), the qPCR results showed a decrease in the expression of marker genes for myogenic differentiation (*p* < 0.01) (Figure 5E,F). The expression of iron metabolism-related genes was detected using qPCR to determine the process of iron ion metabolism (iron delivery to cell: transferrin receptor protein 1 (*TfR1*), hereditary hemochromatosis protein (*Hfe*), and divalent metal transporter1 (*Dmt1*); cellular iron storage: ferritin heavy chain (*FTH*), heme oxygenase 1 (*HO-1*). The iron exporter from the cell, ferroportin1 (*FPN1*)), plays a role in myogenic differentiation. The genes affecting iron homeostasis were the most affected by interference or overexpression (Figure 5G,H). The most significant changes occurred in the IRP-IRE system, which regulates the iron ion homeostasis pathway (*p* < 0.01) (Figure 5I,J). The results of this study showed that STEAP3 overexpression increased intracellular iron ion content and activated the homeostatic IRP-IRE system to regulate the metabolism of intracellular iron ions, thereby promoting myoblast differentiation in black pig myoblasts.

### 3.5. STEAP3 Promotes MuSCs’ Myogenic Differentiation by Regulating the PI3K-AKT Signaling Pathway and Homeostasis

**Figure 6:**
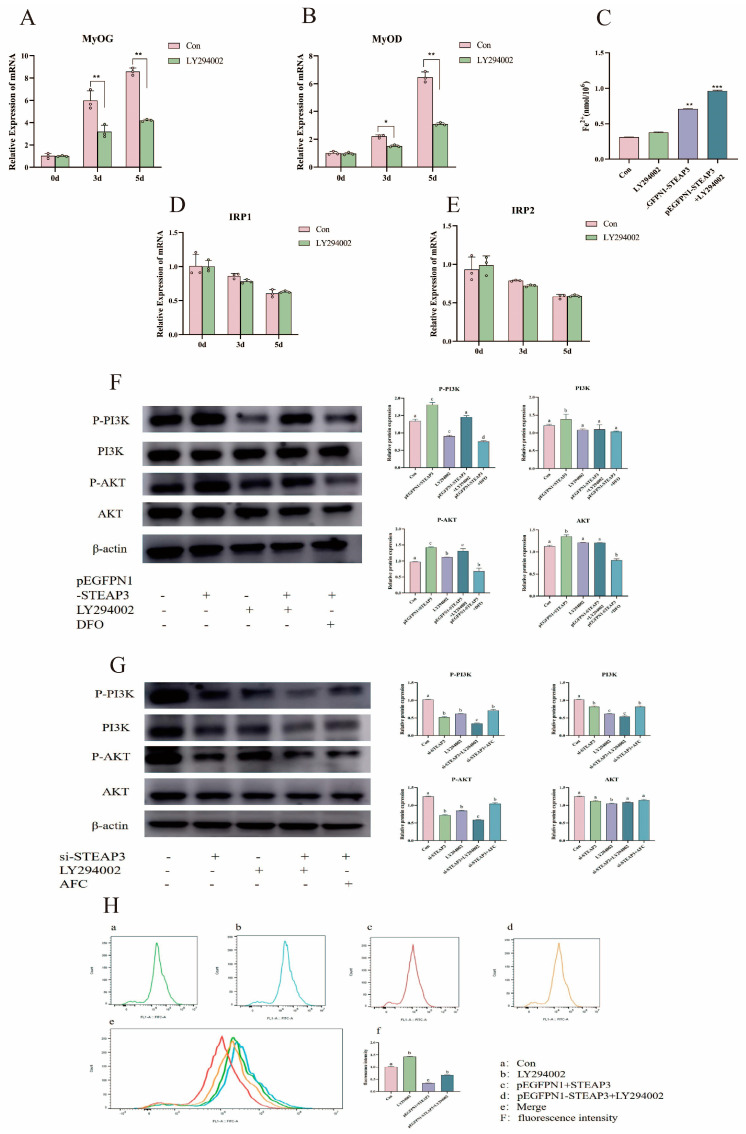
STEAP3 promotes MUSCs’ myogenic differentiation by regulating the PI3K-AKT signaling pathway. (**A**,**B**) Detection of myogenic differentiation genes after the addition of PI3K inhibitors. (**C**) The iron ion content varies under different conditions. (**D**,**E**) Changes in iron homeostasis pathway genes after the addition of PI3K inhibitors. (**F**) Changes in the PI3K-AKT pathway protein after STEAP3 overexpression. (**G**) Changes in the PI3K-AKT pathway protein after STEAP3 interference. (**H**) Changes in intracellular iron pools under different conditions. *n* = 3, * *p* < 0.05, ** *p* < 0.01, *** *p* < 0.01.

**Figure 7:**
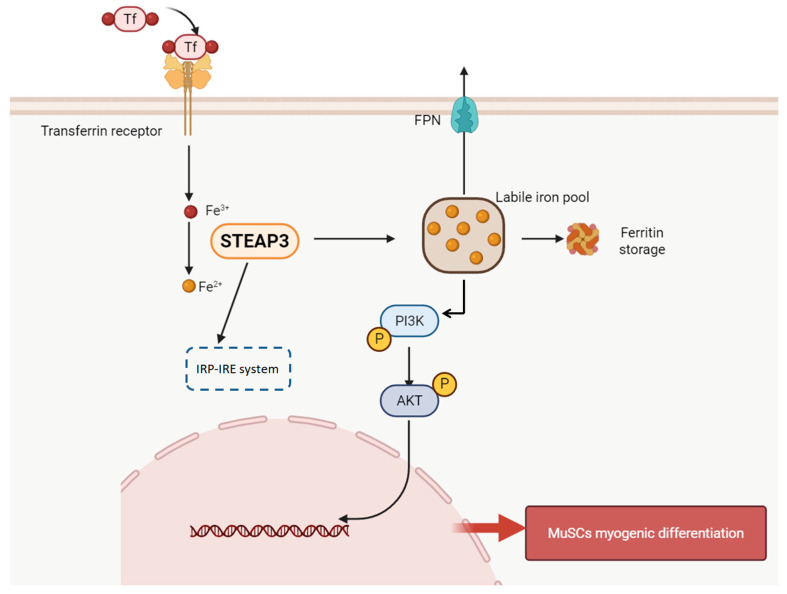
The mechanism of STEAP3 promotes Yunan black pig MuSCs myogenic differentiation.

LY294002 is a specific inhibitor of phosphatidylinositol 3-kinase (PI3K) which inhibits the PI3K/AKT pathway. *MyoG* and *MyoD* expression levels decreased after the addition of LY294002 (*p* < 0.01) (Figure 6A,B). Intracellular Fe^2+^ content significantly increased when STEAP3 was overexpressed and LY294002 was added (*p* < 0.05) (Figure 6C). Western blotting was used to detect the expression of PI3K-AKT pathway proteins. As shown in Figure 6D,E, the IRP-IRPE system has no effect on PI3K/AKT for not changing of IRP1 and IRP2. Then, the expression of P-PI3K and P-AKT increased after STEAP3 overexpression, while the expression of P-PI3K and P-AKT decreased after STEAP3 overexpression and LY294002 addition. However, the expression of P-PI3K and P-AKT decreased after the addition of DFO-chelated iron ions after STEAP3 overexpression. In the STEAP3 knockdown group (Figure 6F), AFC was added to increase the number of iron ions to demonstrate the opposite trend (Figure 6G). Additionally, we used flow cytometry to measure the cell iron pool content and found that Fe (II) promoted PI3K/AKT to help the myogenic differentiation of MuSCs (Figure 6H). Furthermore, we have carried out more experiments for different concentrations (AFC (0, 50, 100 μM); DFO (0, 50, 100 μM)) with MYOG, MYOD, and the PI3K-AKT pathway on the fifth day during myogenic differentiation, as shown in Supplementary Figures S1 and S2. Here, the myogenic differentiation of Yunan black pig MuSCs inhibited both iron overload AFC (0, 50, 100 μM) and depletion DFO (0, 50, 100 μM)) which suggests that myogenic differentiation is based on suitable cellular iron concentration. Furthermore, the PI3K-AKT pathway also changed, showing that cellular iron will promote and then enhance myogenic differentiation, which is in accordance with Figure 6 (Supplementary Figures S1 and S2). The results demonstrated that the excess ferrous ions produced are stored in the iron pool for the body to use at any time. Therefore, STEAP3 firstly promoted the switch between Fe (III) and Fe (II) and enhanced the labile iron pool via the IRP-IRE system during iron ion homeostasis, then activated the PI3K-AKT signaling pathway by forming ferrous ions and promoting the myogenic differentiation of MuSCs (Figure 7).

## 4. Discussion

Myogenesis is the process of skeletal muscle regeneration that begins with the activation of quiescent satellite cells after injury. This is followed by the proliferation, differentiation, and fusion of myoblasts into myotubes, all of which are regulated by complex molecular mechanisms in skeletal muscle [56]. In vitro myogenic differentiation is an important model that has been used to study the mechanism of differentiation in different species, such as pigs, cattle, sheep, and other animals, often using fetal or newborn tissues [57,58,59,60]. In this study, we successfully isolated MuSCs from 3-day-old Yunan black pigs and observed the myotube differentiation marker gene *MyHC* after immunofluorescence staining. The cells were long, shuttle-shaped, and arranged in parallel at day 5 (Figure 1A,B). The qPCR results showed that the expression of *MyoD*, *MyoG*, and *MyH3* increased significantly with an increase in differentiation days (*p* < 0.01) (Figure 1C), which proved that the cells were well differentiated. Therefore, our results are consistent with those of other studies on the isolation, culture, and identification of porcine skeletal MuSCs [49,61]. In summary, we isolated MuSCs from Yunan black pigs and successfully established an in vitro differentiation model. This helped us study the role and exact mechanism of STEAP3 in the myogenic differentiation of Yunan black pig MuSCs in vitro.

The STEAP family, a relatively newly discovered protein, is widely expressed in normal tissues and plays an important role in physiological functions; for example, oxidoreductases participate in the absorption and reduction of iron and copper [62,63]. The STEAP family is also involved in pathological processes through mineral absorption and TP53-regulating transcription of cell death genes and ferroptosis, especially in cancer [64,65]. Although STEAP3 has been detected in skeletal muscles, there are few reports on this condition [28,30]. We observed that STEAP3 expression was higher than that of other STEAP family members, such as STEAP1, STEAP2, and STEAP4, during the myogenic differentiation of Yunan black pig MuSCs in vitro (Figure 2A–D). Skeletal muscle IHC and Western blotting showed that STEAP3 was mainly distributed in the cell membrane of myofibrils, and the distribution of STEAP3 was significantly higher in the skeletal muscle at 6 months (Figure 2E–H). These results suggested that STEAP3 plays an important role in myogenic differentiation. Furthermore, myogenic differentiation is a complex stage in which the myogenic regulatory factors MYF5, MRF4, and MyoG overcome inhibitory factors to prevent premature myogenic commitment and withdrawal from the cell cycle [66,67]. Therefore, MyoD and MyoG expression can induce skeletal muscle differentiation and are important markers in fibroblasts, which are of mesodermal derivation like myoblasts and derived from the two other germ layers in some cell types [6,7]. The myotubes then accumulate contractile sarcomeric proteins such as actin, myosin heavy chain, tropomyosin, and desmin to form skeletal muscle fibers that play key roles in locomotion, postural maintenance, respiration, and thermogenesis [5]. We obtained the si-STEAP3-treated and STEAP3-overexpressed groups in this study (Figure 3A–D and Figure 4A–D). Furthermore, we found that the expression of the myogenic differentiation marker genes MyoG and MyoD and the number of myotubes (marked by MyHC) formed by the differentiation of cells in the si-STEAP3-treated group were significantly decreased compared to that in the control group (Figure 3G–J). Conversely, when STEAP3 was highly expressed in myoblasts, the expression levels of MyoG and MyoD increased. Additionally, high expression of STEAP3 leads to enhanced differentiation of myoblast cells, and immunofluorescence staining for MyHCs showed a significant increase in the number of multinucleated myotubes formed (Figure 4G–J). Collectively, these results indicated that STEAP3 promotes myoblast differentiation.

Iron is a vital element in the body and has been reported mainly in hemoglobin (60–70%) as it carries and circulates oxygen throughout the body. Iron can be found in myoglobin in skeletal muscles and plays a key role in muscle cell development [68,69,70]. Several studies have shown that iron doses should be carefully controlled to achieve beneficial effects in skeletal muscle cells. Therefore, both iron deficiency and high doses of iron ions have been reported to negatively affect cells and are associated with skeletal muscle diseases and frailty. For instance, iron deficiency (ID) is a widespread condition concomitant with diseases that result in systemic dysfunction of target tissues, including skeletal muscle [71]. Furthermore, iron overload suppresses the differentiation of C2C12 myoblast cells and inhibits myogenesis via oxidative stress accompanied by membrane lipid peroxidation and muscle wasting, leading to an imbalance in skeletal muscle homeostasis, even in old mice [72,73]. Fe-bound transferrin promoted myogenic cell growth and myotube formation under 10–100 μm or 5–500 µM Fe ions in the cell culture medium [74,75]. We also found that intracellular Fe^2+^ content increased significantly with an increase in the number of differentiation days (Figure 5A–C). A decrease in the expression of marker genes for myoblast differentiation was observed after iron overload (AFC) and iron depletion (DFO), demonstrating that an appropriate amount of iron ions indeed affects myoblast differentiation (Figure 5D). Next, iron acquisition, storage, and/or efflux must be carefully orchestrated to achieve iron homeostasis under different conditions to avoid intracellular iron levels that are too low or too high. Therefore, multiple regulatory systems operate at every level of gene regulation to control iron balance [76,77]. The IRP-IRE regulatory network is mainly responsible for iron homeostasis and regulates the post-transcriptional expression of key iron metabolism genes by binding with nanomolar affinity to cis-regulatory iron-responsive elements (IREs) using stem-loop RNA structures located in the UTR of target transcripts [78,79,80]. We examined the expression of genes related to iron ion uptake, homeostasis, and efflux during iron metabolism to determine the role of STEAP3 in iron ion metabolism and myogenic differentiation. The expression of genes related to iron ion homeostasis changed the most when STEAP3 was disrupted or overexpressed. The IRP-IRE system played a major role in the classical pathway that regulates iron ion homeostasis (Figure 5G–J). The results of this study showed that the overexpression of STEAP3 increased intracellular iron ion content and activated the homeostatic IRP-IRE system to regulate the metabolism of intracellular iron ions, thus promoting myoblast differentiation in Yunan black pig MuSCs.

Next, we clarified how STEAP3 regulates the myoblasts of Yunan black pig MuSCs. The PI3K/AKT pathway appears to act as an effector of different factors that can induce myogenic differentiation under stage-specific conditions [8,9]. In our study, *MyoG* and *MyoD* notably decreased after treatment with LY294002 (a specific inhibitor of PI3K) during myogenic differentiation on days 3 and 5 compared to that in the control group (Figure 6A–C). Furthermore, the pEGFPN1-STEAP3 group (overexpression) significantly activated the PI3K/AKT pathway for p-PI3K and p-AKT expression but decreased and was also inhibited when treated with LY294002 (a specific inhibitor of PI3K). p-PI3K and p-AKT expression decreased in the si-STEAP3 group where the STEAPs expression was inhibited (Figure 6F). Therefore, these results are consistent with those of a previous study and suggest that PI3K/AKT is involved in the pathway of STEAP3-regulated Yunan black pigs. We then examined the relationship between Fe ions and PI3K/AKT. As shown in Figure 6G, p-PI3K and p-AKT decreased or increased in the DFO-treated group with iron depletion or the AFC-treated group with iron overload compared to that in the control, pEGFPN1-STEAP3, and si-STEAP3 groups, respectively. Thus, our results indicated that iron ions activate the PI3K/AKT pathway. The phosphorylation levels of AKT (p-AKT) were strongly increased in osteoblast cells treated with ferric ammonium citrate (FAC) 100 μmol/L as an iron overload condition [81]. Furthermore, there were simultaneous increases in PI3K and AKT phosphorylation of mouse hippocampal neurons (HT22) or cerebral cortex synaptic endings from rats treated with different concentrations of Fe^2+^ (25–200 μm) for 24 h [82,83]. However, iron overload significantly inhibits the phosphorylation of PI3K and AKT in MC3T3-E1 cells [84]. Furthermore, ammonium iron citrate (FA group) increased intracellular ROS levels and had a negative effect on p-PI3K/PI3K and p-Akt/Akt compared to that in the control group (*p* < 0.05) in C2C12 cells [85]. Therefore, the effect of iron on the PI3K/AKT pathway is based on intracellular conditions and depends on the iron ion concentration. Additionally, cellular iron homeostatic metabolism is mainly regulated by IRPs, which are categorized into IRP1 and IRP2 as an imbalance in intracellular iron metabolism [34,35,40]. We also used flow cytometry to measure the cell iron pool content and detected the expression of IRP1 and IRP2 using PCR, demonstrating that the excess ferrous ions produced are stored in the iron pool for the body to use at any time (Figure 6H). In summary, STEAP3 activates the PI3K-AKT signaling pathway by forming ferrous ions, thereby promoting myogenic differentiation of satellite cells.

## 5. Conclusions

In conclusion, this study found that STEAP3 is involved in myogenic differentiation based on the Yunan black pig MuSCs model in vitro as the differentiation of cells from the si-STEAP3-treated group was significantly decreased but increased in the STEAP3 overexpression group compared to that in the control group. Furthermore, STEAP3 firstly promoted the switch between Fe (III) and Fe (II) and enhanced the labile iron pool via the IRP-IRE system during iron ion homeostasis, then activated the PI3K-AKT signaling pathway by forming ferrous ions, promoting myogenic differentiation of MuSCs. The results of this study showed that STEAP3 overexpression increased intracellular iron ion content and activated the homeostatic IRP-IRE system to regulate intracellular iron ion metabolism.

## Figures and Tables

**Table 1:** Sequences PCR primers used for RT-PCR.

Gene	GenBank	Primer Sequences	Product Length/bp	Annealing Temperature/°C
*β-Actin*	XM_021086047.1	F: CCACTCCGCCAGCACAGG R: CATCGTCGCCCGCAAAGC	147	60
*STEAP3*	XM_003359436.4	F: TGAGGAGGAAGTCTGGCGGATG R: CAATGGATGGCAGTGAGGTAGCAG	125	61
*STEAP1*	NM_214305.2	F: ATCCTGGCTCTACTGGCTGTGAC R: ATTGTGCCCAGCAGAAGGGAAAC	116	57
*STEAP2*	XM_003357423.4	F: CTCCATCCCTTCCGTGAGCAATG R: GAGCAGAGCGACATAGCCAAGTG	82	59
*STEAP4*	NM_001166489.1	F: TGTGTACGGCGGGAGGAGATTC R: CGGCAGGATGAGGATGAACTTGAC	124	60
*MyOG*	NM_001012406.1	F: AAACTACCTGCCCGTCCACCTC R: GGTCCCCAGCCCCTTATCTTCC	73	62
*MyOD*	NM_001002824.1	F: AACTGTTCCGACGGCATGAT R: TCGCTGTAATAGGTGCCGTC	86	57
*MyH3*	XM_013981330.2	F: TGCTTAGCGCGTAGGCTTACGA R: TGACGAAGGCCCTACGCAATG	97	57
*TfR1*	NM_214001.1	F: ATTCCCCGTTGTTGAGGCAGAC R: TGACTGAGATGGCGGAAACTGAG	118	58
*Hfe*	XM_021098424.1	F: GCCTACCTGGAGCGGGACTG R: AGAGCCTGACAGCGTAGAGTGG	143	58
*HO-1*	NM_001004027.1	F: GGCATCCGACATCCGCAAGAG R: CACCTGGGAGAGGACGCTGAG	100	60
*Dmt1*	XM_021081706.1	F: TGGAGGATCGCAGGCGGTATC R: AGCCACCACATACAACACCACATG	108	59
*FTH*	NM_213975.1	F: TGCCAAATACTTTCTCCACCAATCTC R: CCCGCTCTCCCAGTCATCAC	136	57
*FPn1*	XM_003483701.4	F: GCAACAGCGGCAGCGGTAG R: GCATCCTCCCTGGCGGTTTTG	91	58
*IRP1*	XM_003357729.3	F: CTGTGGGAATGTTTCGGGAT R: CCACTGCAGCAAGGCACTAC	112	57
*IRP2*	NM_001167781.1	F: TGGTCATTGCTGCCGTTATC R: TGTAACCATCCCACTGCCTG	109	59

## Data Availability

The original contributions presented in this study are included in the article/Supplementary Materials. Further inquiries can be directed to the corresponding authors.

## Supplementary Materials

The following supporting information can be downloaded at: https://www.mdpi.com/article/10.3390/cells14090656/s1, Figure S1. Concentration screening of AFC and DFO, and the influence of different concentrations of AFC on the pathway. Figure S2. The effect of adding DFO on the PI3K-AKT signaling pathway.
