# Supplementary material for: The Six-Transmembrane Epithelial Antigen of the Prostate (STEAP) 3 Regulates the Myogenic Differentiation of Yunan Black Pig Muscle Satellite Cells (MuSCs) In Vitro via Iron Homeostasis and the PI3K/AKT Pathway
Source: Cells. 2025 Apr 29;14(9):656. doi: 10.3390/cells14090656 (PMC12071230; doi:10.3390/cells14090656)
Supplement: Supplementary file 1 [file cells-14-00656-s001.zip › cells-3573170-supplementary.pdf]

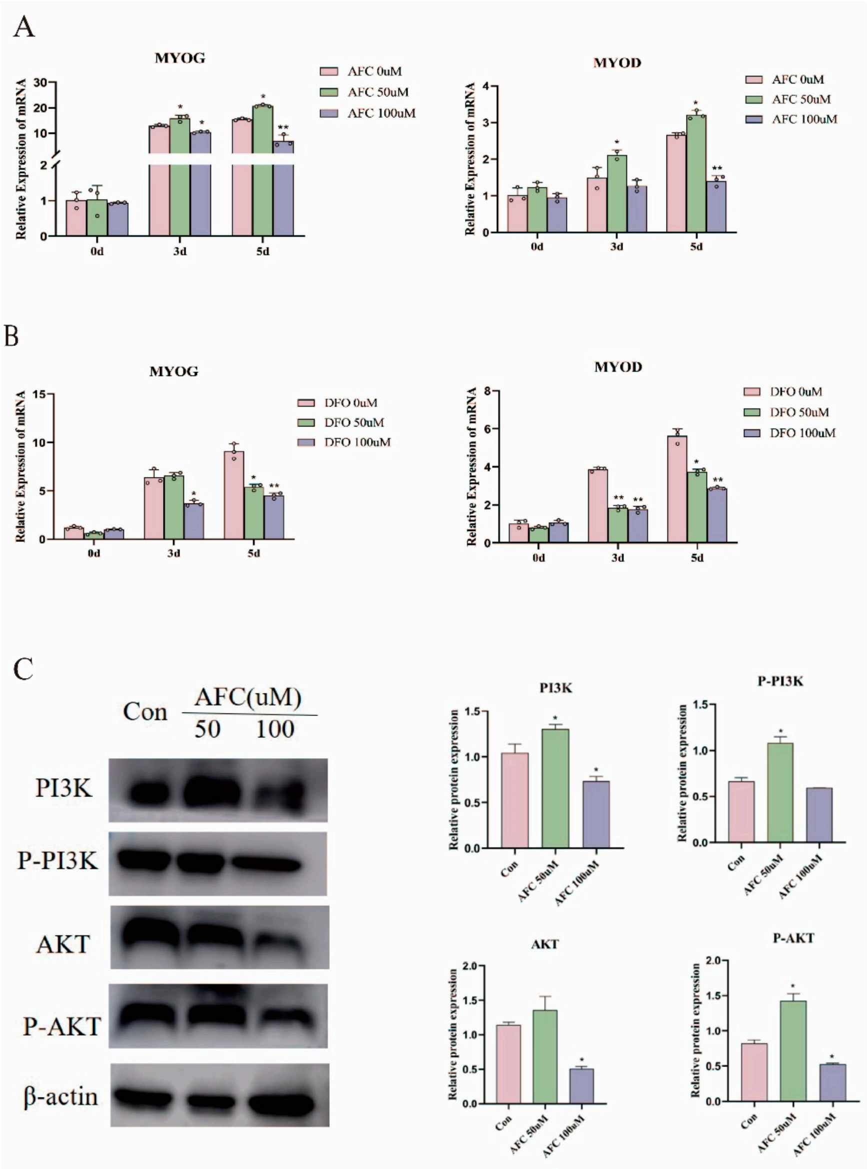

Figure S1. Concentration screening of AFC and DFO, and the influence of different concentrations of AFC on the pathway. (A) The changes of MyOG and MyOD under different concentrations of AFC (B) The changes of MyOG and MyOD under different concentrations of DFO (C)The changes of the PI3K-AKT pathway under different concentrations of AFC.n=3,* P<0.05, **P<0.01,*** P<0.01.

A

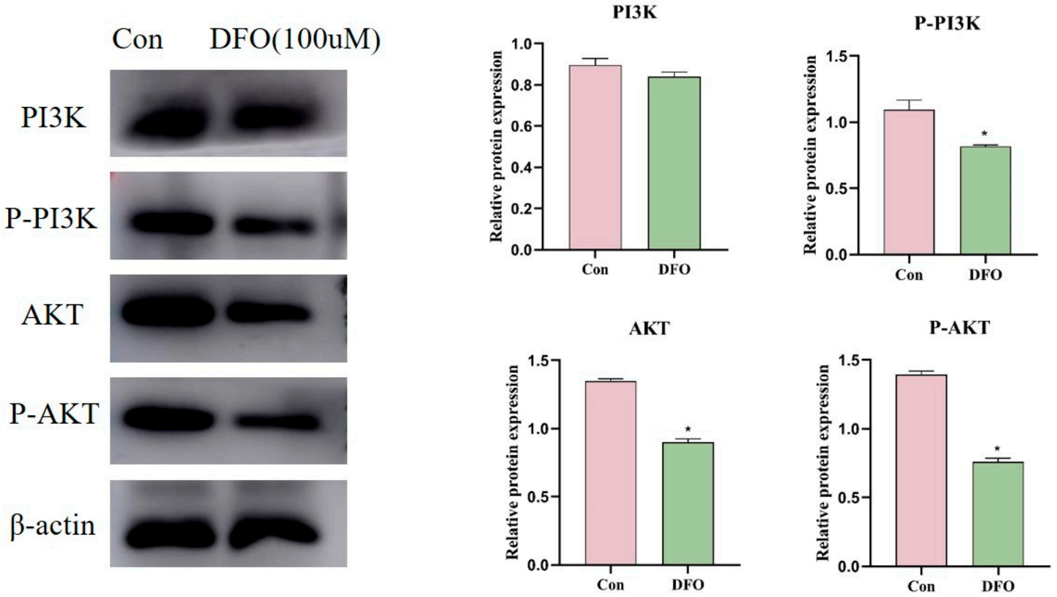

Figure S2. The effect of adding DFO on the PI3K-AKT signaling pathway. n=3,* P<0.05.
